# Health inequities: lower socio-economic conditions and higher incidences of intestinal parasites

**DOI:** 10.1186/1471-2458-7-342

**Published:** 2007-11-27

**Authors:** İpek Östan, Ali A Kilimcioğlu, Nogay Girginkardeşler, Beyhan C Özyurt, M Emin Limoncu, Ülgen Z Ok

**Affiliations:** 1 Celal Bayar University, Vocational School of Health Services, Manisa, Turkey; 2 Celal Bayar University, Faculty of Medicine, Department of Parasitology, Manisa, Turkey; 3 Celal Bayar University, Faculty of Medicine, Department of Public Health, Manisa, Turkey

## Abstract

**Background:**

Intestinal parasitic infections affect child health and development and slow down growth, while reducing adults' productivity and work capacity. The aim of the present study was to determine and compare the incidences of intestinal parasitic infections and the socio-economic status of two near primary school children in Manisa, a western city of Turkey.

**Methods:**

A total of 352 children were involved a questionnaire study from a private school (Ülkem Primary School – ÜPS, 116 children) and a community-based school (Şehzadeler Primary School – ŞPS, 236 children). Of these, stool samples could be obtained from a total of 294 students; 97 (83.6%) from ÜPS, and 197 (83.5%) from ŞPS. The wet mount preparations of the stool samples were examined; samples were also fixed in polyvinyl alcohol and examined with modified formalin ethyl acetate sedimentation and trichrome staining techniques. Data were analyzed using SPSS for Windows version 10.0. The chi-squared test was used for the analytic assessment.

**Results:**

The percentages of the students found to be infected with intestinal parasites, were 78 (39.6%) and 13 (13.4%) in ŞPS and ÜPS, respectively. Totally 91 (31.0%) of the students from both schools were found to be infected with at least one intestinal parasite. *Giardia lamblia *was found to be the most common pathogenic intestinal parasite and *Blastocystis hominis *was prevalent independently from the hygienic conditions. The factors which significantly (*p *< 0.05) increase the incidence of intestinal parasites were uneducated and unemployed mother, lower social status of father, living in crowded houses with insufficient indoor spaces, using the tap water as drinking water, and living at shanty areas.

**Conclusion:**

Intestinal parasitic infections in school children were found to be a public health problem that increased due to lower socio-economic conditions. We conclude that organization of education seminars including the topics such as prevention of the infectious diseases, improving general hygienic conditions, and application of supportive programs for the parents may be suggested not only to reduce intestinal parasitic infections, but also to elevate the socio-cultural levels.

## Background

The burden of diseases associated with intestinal parasitic infections is enormous. About two billion people are affected worldwide, of whom 300 million suffer from associated severe morbidity [1]. Indirect morbidity is particularly important in children with parasitic infections, ranging from malnutrition, anaemia, growth retardation, irritability and cognitive impairment to increased susceptibility to other infections and acute complications [2]. Epidemiological studies carried out in different countries have shown that the socio-economic level of the society may affect the incidence of intestinal parasites; control strategies of local managements involving improved infra-structure for both drinking water and sewage system, education of the society to improve personal hygiene and sanitation have been related to reduced incidence of intestinal parasites [1,3].

To evaluate the effect of socio-economic conditions on the incidence of intestinal parasites, stool samples of the students in two primary schools, which are very close to each other in the city center of Manisa, were examined. The students of the two schools had different socio-economic conditions and the schools had different hygienic circumstances.

## Methods

### Study population

Manisa is located in western Turkey and has become an important industrial province during the last decades depending on its rapidly increased population of 1.260.169, according to the census carried out in 2000.

The two primary schools, Şehzadeler Primary School (ŞPS) and Ülkem Primary School (ÜPS) are both full-time schools located in the same district of Manisa. ŞPS is a community-based school and about half of the students live in shanty areas. The students mostly have their lunch at home or buy something to eat from the neighborhood groceries. The average number of the students in the classrooms was 30. The number of staff for cleaning in the school was insufficient. The number and hygienic conditions of toilets were not conforming to the number and conditions of the criteria published by the Turkish Standard Institute, which required one toilet cabinet for 25 girls and 40 boys, one urinal for 25 boys and one tap for ≤25 children [4].

The soap and toilet paper were not available in the toilets. The school was free of charge for the students. ÜPS is a private school and the students of ÜPS live in wealthy districts with an improved infrastructure. The annual fee of this school was almost 4,000 USD per student. The average number of students in the classrooms was 25. The sanitary and the hygienic conditions in the school were conforming to every health regulation. The school had a contracted food supplier company for the lunch; therefore the students and the teachers had lunch in the refectory of the school under good sanitary and hygienic conditions. The number and the conditions of the toilets in the school were conforming to the criteria specified by the Turkish Standards Institute strictly [4]. The soap and toilet papers were always available in the toilets.

All children in grade 3, 4 and 5 in both schools were enrolled in the study. In each grade, there were three classes in ŞPS and two classes in ÜPS; therefore, the number of students examined was higher in ŞPS than ÜPS. A questionnaire was applied and totally 352 children, 236 from ŞPS and 116 from ÜPS, were involved in the study. Of these, stool samples were obtained from a total of 294 students, 197 (83.5%) and 97 (83.6%) from ŞPS and ÜPS, respectively. The study was carried out between November and December 2006. Before the study, the permissions were obtained from the Provincial Education Administration of Manisa and School Directorships. The study was approved by Scientific Researches Ethics Committee of Celal Bayar University.

In order to inform the students and their families about the content of the study, a leaflet was given to all children and the procedure for stool sample collection was explained. Sanitary and hygienic conditions and water supply of the schools were also reviewed.

### Data collection

The questionnaire consisting of 25 questions was applied to the students face to face in the classroom by the public health specialist, accompanied by the teachers. The socio-demographic information, the social status of fathers (questioned in terms of employment, not income) specifications of the houses, the hygienic habits of the student and the health complaints during the last six months were asked in the questionnaire.

### Collection and examination of stool samples

A plastic container marked with an identification number was given and one fresh stool sample was collected from each child. Wet mount preparations of the stool samples were immediately examined with x40 objectives in the school laboratories. The stool samples were also fixed in polyvinyl alcohol (PVA) fixative solution and transported to the laboratory in the Department of Parasitology. Modified formalin ethyl acetate sedimentation and trichrome staining techniques were performed from each fixed stool sample and all samples were examined by two experienced parasitologists, who were blinded to specimen identification. The prescriptions were given to the students found to be infected with pathogen parasites and their families were informed about the treatment.

### Statistical analysis

Data were analyzed using SPSS for Windows version 10.0. The chi-squared test was used for the analytic assessment. The differences were considered to be statistically significant when the p-value was less than 0.05.

## Results

A total of 294 children, aged between 9–12 years, (mean = 9.76 ± 0.99) were examined for intestinal parasites. The parasites detected in the stool samples obtained from the students of both schools are given in Table 1.

**Table 1:** Distribution of the intestinal parasites in the students of two primary schools

**Parasites**	Şehzadeler (ŞPS) (n = 197)	Ülkem (ÜPS) (n = 97)	Total (n = 294)
**Pathogenic spp.**	n (%)	n (%	n (%)
*Giardia intestinalis*	27 (13.7)	0 (0)	27 (9.2)
*Dientamoeba fragilis*	8 (4.0)	2 (2.1)	10 (3.4)
*Enterobius vermicularis*	3 (1.5)	0 (0)	3 (1.0)
*Hymenolepis nana*	1 (0.5)	0 (0)	1 (0.3)
**Suspicious pathogenic spp**.			
*Blastocystis hominis*	30(15.2)	12 (12.4)	42 (14.2)
*Entamoeba histolytica/dispar*	3 (1.5)	1 (1.4)	4 (1.3)
**Non-pathogenic spp.**			
*Entamoeba coli*	17 (9.0)	1 (1.4)	18(6.2)
*Endolimax nana*	11 (5.6)	0 (0)	11(3.7)
*Iodomoeba bütschlii*	2 (1.0)	0 (0)	2 (0.6)

The numbers of children with and without parasites in two schools are shown in Table 2.

**Table 2:** The numbers of children with and without parasites in two schools^a^

	Şehzadeler (ŞPS)	Ülkem (ÜPS)	Total
	n (%)	n (%)	n (%)

Parasites +	78 (39.6)	13 (13.4)	91 (31.0)
Parasites -	119 (60.4)	84 (86.6)	203 (69.0)
Total	197 (100)	97 (100)	294 (100)

^a^Some of children have more than one parasite.

The comparison of the socio-demographic characters, house specifications and hygiene habits found to be statistically significant in two schools are given in Table 3.

**Table 3:** Comparison of two primary schools based on statistically significant characteristics

**Characteristics**	Şehzadeler (ŞPS)	Ülkem (ÜPS)	
**Socio-demographic characteristics**	n (%)	n (%)	*p *value

Residence (n = 287)			
Shanty area	79 (40.7)	0 (0)	
City	115 (59.3)	93 (100)	<0.001

Education of mother (n = 284)			
Uneducated/primary school Incomplete	63 (32.6)	0 (0)	<0.001
Primary school	89 (46.1)	5 (5.5)	
High school and more	41 (21.2)	86 (91)	

Father's social status^a ^(n = 255)			
High	52 (30.6)	84 (98.8)	<0.001
Low	118 (69.4)	1 (1.2)	

Mother's employment (n = 293)			
Employed	23 (11.7)	56 (57.7)	< 0.001
Unemployed	173 (88.3)	41 (42.3)	

**Housing conditions**			

Owner (n = 294)			
Yes	123 (62.4)	77 (79.4)	0.03
No	74 (37.6)	20 (20.6)	

Each student has his/her own bedroom (n = 291)			
Yes	45 (23.1)	85 (88.5)	< 0.001
No	150 (76.9)	11 (11.5)	

Number of person living in house (n = 292)			
≤4 (n = 47)	16 (8.1)	31 (32.0)	< 0.001
>4 (n = 247)	181 (91.9)	66 (68.0)	

Number of brother/sister (n = 294)			
≤2	91 (46.2)	87 (89.7)	< 0.001
>2	100 (53.8)	10 (10.3)	

Drinking water (n = 288)			
Treated	176 (92.1)	24 (24.7)	< 0.001
Untreated	15 (7.9)	73 (75.3)	

Washbasin in toilet (n = 290)			
Yes	18 (61.1)	97 (100)	<0.001
No	75 (38.9)	0 (0)	

**Hygienic habits**			

Washing hands (n = 293)			
Only water	117 (59.7)	22 (22.6)	< 0.001
With soap and water	79 (40.3)	75 (77.3)	

^a^The indicators of father's social status: Low social level: unemployment, working at unqualified jobs, working at temporary jobs. High social level: employee, employer, employee based on high wage, craftsman

Statistically significant relations (*p *<0.05) between the intestinal parasites and the socio-demographic characteristics of the students, the specifications of the houses where they live and the hygienic habits, are given in Table 4. No significant relation was found between the incidence of parasites and the sex of the students, the number of the bedroom in their houses, the habit of using toilet paper. The incidence of parasitic infection was found to be decreased from 45.8% to 23.5%, when the education level of the father was higher although the relation was not found to be statistically significant.

**Table 4:** Statistically significant correlations between characteristics and intestinal parasites

**Characteristics**	Overall parasites		
	Positive n (%)	Negative n (%)	*p *value

**Socio-demographic characteristics**			

Residence			
Shanty area (n = 79)	32 (40.5)	47 (59.5)	0.03
City (n = 208)	57 (24.4)	151 (72.6)	

Education of mother			
Uneducated/primary school Incomplete (n = 63)	29 (46.0)	34 (54.0)	<0.001
Primary school (n = 94)	36 (38.3)	58 (61.7)	
High school and more (n = 127)	25 (19.7)	102 (80.3)	

Father's social status^a^			
High (n = 136)	27 (19.9)	109 (80.1)	<0.001
Low (n = 119)	54 (45.4)	65 (54.6)	

Mother's employment			
Employed (n = 214)	79 (36.9)	135 (63.1)	<0.001
Unemployed (n = 79)	12 (15.2)	67 (84.8)	

Age			
9 (n = 100)	30 (30.0)	70 (70.0)	0.04
10 (n = 120)	30 (25.0)	90 (75.0)	
11–12 (n = 74)	31 (41.9)	43 (58.1)	

**Housing conditions**			

Each student has his/her own bedroom			
Yes (n = 130)	25 (19.2)	105 (80.8)	<0.001
No (n = 161)	66 (41.0)	95 (59.0)	

Number of person living in house			
≤4 (n = 47)	8 (17.0)	39 (83.0)	0.02
>4 (n = 247)	83 (33.6)	164 (66.4)	

Number of brother/sister			
≤2 (n = 178)	38 (21.3)	140 (78.7)	<0.001
>2 (n = 116)	53 (45.7)	63 (54.3)	

Drinking water			
Treated (n = 88)	14 (15.9)	74 (84.1)	<0.001
Untreated (n = 200)	76 (38.0)	124 (62.0)	

Washbasin in toilet			
Yes (n = 215)	53 (24.7)	162 (75.3)	<0.001
No (n = 75)	37 (49.3)	38 (50.7)	

**Hygienic habits**			

Washing hands (n = 293)			
Only water	52 (37.4)	87 (62.6)	0.01
With soap and water	38 (24.7)	116 (75.3)	

^a^The indicators of father's social status: Low social level: unemployment, working at unqualified jobs, working at temporary jobs. High social level: employee, employer, employee based on high wage, craftsman

Of the complaints of the children with parasitic infections during last six months, only abdominal pain and anal pruritus were statistically significant Of the 199 students with abdominal pain, 74 (37.2%) were positive for intestinal parasites, while parasites were detected in only 17 of 94 students (18.1%) without abdominal pain. Twenty-eight of 67 (41.8%) children with anal pruritus and 63 of 227 (27.8%) children without anal pruritus were found to be positive for intestinal parasites. The relation between intestinal parasites and other complaints such as diarrhoea, nausea, vomiting and lack of appetite were not significant.

## Discussion

Today, Manisa is one of the leading agricultural and industrial cities of Turkey due to the rapid development in its economic structure during the last decades. This rapid growth brought in part some new problems related to the increasing population due to immigration and inadequate infra-structure. New districts with shanty houses and inadequate infrastructure were established on the outskirts of the city center in recent years, where the immigrant families moved. The nutrition, sanitary conditions of the families were insufficient and indoor space of the shanty houses for each household member was limited due to the high number of children [3,5]. It has been observed that the immigrant families who moved to the western side and metropolitans have kept on their habits. Therefore, the frequency of intestinal parasites was found to be significantly higher in the families living in the shanty areas of the cities than the families living in the other sections of the cities [5]. A recent comparison of the intestinal parasites in school children of two neighbouring villages of Manisa, one with immigrants from eastern Anatolia and the other with local people, has revealed higher prevalence rates in the village of the immigrants [6]. Intestinal parasites are known to be related to hygiene behaviour closely, the members of the family can easily be infected from each other. The parasites that are transmitted through faecal-oral route may be an important indicator of socio-economic level. Evaluation of the intestinal parasites detected in our study according to their route of transmission revealed that most of the infections were transmitted directly via the faecal-oral route. Pathogen, possible pathogen and/or non-pathogen intestinal parasites were detected in 31.0% of the children, between 9–12 ages. Similarly, the incidence of cases positive for intestinal parasite was found to be 31.8% in primary school students between 7–14 ages in Aydın, another city in western Turkey [7]. Among pathogen parasites, the incidence of *Giardia lamblia *(also called *Giardia intestinalis, Giardia duodenalis*) was found at the highest level (13.7%) in ŞPS; but surprisingly this parasite was not detected in ÜPS. The incidences of the non-pathogenic *Entamoeba coli *and *Endolimax nana *were also found to be significantly higher in ŞPS than ÜPS and this data posed the question whether some parasites were affected more than others by socio-economic conditions.

There are some discrepancies among the parasite species detected between the eastern and western parts of Turkey. The protozoa,*G. lamblia *and *Entamoeba coli *are the most frequently observed parasites and the incidence of geohelminths such as *Ascaris lumbricoides *and *Trichuris trichiura *is very low in western regions [7,8].

The incidence of *Blastocystis hominis *was found to be highest (14.2%) among all parasites. The pathogenicity of *B. hominis *is still controversial, but it is usually regarded as a potential pathogen and the application of trichrome staining method raises the incidence of this organism [9]. The incidences of this organism were 15.2% and 12.4%, in ŞPS and ÜPS, respectively. The data that the incidence of *B. hominis *was not affected from the socio-economic level suggested that this organism would possibly remain prevalent in future, although better hygienic conditions might be reached.

Another interesting finding was the low incidence of helminthic infections such as *Enterobius vermicularis *and *Hymenolepis nana *in both schools. The low incidence of *E. vermicularis *may be explained by the lack of application of cellophane tape method and the low incidence of other helminths might be related to recovery of hygienic conditions. The similar results were obtained in the studies carried out in Manisa and Aydın as well [6,7]. In contrary, the incidences of *A. lumbricoides *and *T. trichiura *was reported between 4.1–41.4% and 4.9–5.2%, respectively, in two studies carried out in Southeast Turkey [10,11]. Geohelminths were less frequently reported in western than eastern regions probably-due to insufficient sewage system and the application of stool wastes as fertilizer in the vegetable and fruit gardens [7,12].

The significantly higher detection of the parasites in the children living in shanty areas than the ones living in the centrum and other districts was also observed in similar studies [6,11,13]. Among the students in ŞPS, 40.7% were found to be positive for intestinal parasites and 39.6% were living in shanty area. This data suggested that the incidence of intestinal parasites was not only related to the areas where they live, but also with the socio-demographic features and other factors such as cleaning and health habits of the families.

No significant relation was detected between the parasitic infections and the sex of the students. However, a significant relation was found between older students and increased parasite frequencies as in the similar studies [6,11,13]. No significant relation was determined between the presence of intestinal parasites and the education level of the father; but the education level of the mother was correlated with lower parasite levels. There are studies reporting the same result [13], but relation between education level of the father and incidence of intestinal parasites was also reported [14]. The education levels of mothers who had graduated from high school or higher were found to be 21.2% and 91.0%, in ŞPS and ÜPS, respectively. The higher education level of the mother leads to lower incidence of intestinal parasitic infection in children. In addition, there was statistically significant difference between prevalence of parasitic infections among children whose mothers had regular jobs and could spare time for child care only after work and children whose mothers had no regular jobs and could spare more time to child care (36.9% and 15.2%, respectively).

It is reported that the risk of transmission of parasitic infections becomes higher in the crowded families due to close contact [3]. We found that the parasite incidence in students who have their own bedroom was significantly lower. Increased number of household members was related with the higher incidence of intestinal parasites. The ratios of having more than two brothers or sisters were 53.8% in ŞPS and 10.3% in ÜPS. It was also determined that the brother/sister averages in students in both schools were 2.2 (sd ± 2.0) with intestinal parasites and 1.46 (sd ± 1.69) without intestinal parasites. This result was statistically significant.

The students who drink the tap water were found to have higher level of parasites than the students who drink commercial drinking water in demijohn (38.0% and 15.9%, respectively); and this result has supported the suggestion that the tap water network of Manisa should be revised [15]. In a study carried out in Argentine, it was determined that intestinal parasite frequencies detected in various socio-cultural areas were related to contaminated water resources by the parasites, as well as the insufficient health conditions [16]. Surprisingly, it was suggested that no correlation was seen between the reliable drinking water and parasitic infections in another study carried out in Mexico [13].

We found no relation between the intestinal parasites and the habit of washing anal region with hand or using the toilet paper. This result is different from the findings of Okyay et al [7] and may be explained by the fact that washing the anal region with hand is common at each social level in the society.

Trichrome staining method was found to be beneficial to detect particularly *Dientamoeba fragilis *trophozoites and formalin ethyl acetate concentration method was effective in detection of protozoa cysts. It was considered that when the appropriate diagnosis methods was used, the incidence of *D. fragilis*, an intestinal pathogen protozoon only be detected by using trichrome staining method, would be much higher. None of the samples found to be negative by both trichrome staining and formalin ethyl acetate concentration methods was positive by direct wet mount preparations for intestinal parasites. This result supports the view that direct wet mount is not necessary when trichrome staining and formalin ethyl acetate concentration methods were performed [17,18].

As a sole fixative, PVA gave good results with both trichrome staining and formalin ethyl acetate concentration methods. Thus, we agree with the authors who strongly recommended this fixative in the field works, especially when the fresh stool is difficult to bring to the laboratory [18-20].

## Conclusion

Intestinal parasitic infections in school children were found to be a problem of public health that is on the rise due to lower socio-economic conditions. The factors which significantly increase the incidence of intestinal parasites were uneducated mother, low social status of father, living in crowded houses with insufficient indoor spaces, using the tap water as drinking water, and living at shanty areas.

Organization of education seminars including the topics such as prevention of the infectious diseases, improving general hygienic conditions and application of supportive programs for the parents may be suggested not only to reduce intestinal parasitic infections, but also to elevate the socio-cultural levels.

*G. lamblia *was found to be the most common pathogenic intestinal parasite. Although its pathogenicity is controversial, *B. hominis *was found to be prevalent independently from the hygienic conditions in both schools and may be predicted to remain prevalent in future. Our data also suggested that PVA fixative was effective and direct wet mount preparations may not be necessary when trichrome staining and formalin ethyl acetate concentration methods were used together.

## Competing interests

The author(s) declare that they have no competing interests.

## Authors' contributions

iÖ planned the research, organized the work in the schools, performed the sampling and wrote draft of the manuscript. AAK and NG performed parasitic examinations, contributed discussing the results and writing manuscript. BCÖ performed the sampling, data input and statistical analyzes. MEL helped parasitic examinations and prepared the tables. ÜZO coordinated the study, contributed discussing the results and revising the final manuscript.

## Pre-publication history

The pre-publication history for this paper can be accessed here:


